# Targeted Delivery of the Mitochondrial Target Domain of Noxa to Tumor Tissue via Synthetic Secretion System in *E. coli*

**DOI:** 10.3389/fbioe.2020.00840

**Published:** 2020-07-17

**Authors:** Daejin Lim, Woong Chae Jung, Jae-Ho Jeong, Miryoung Song

**Affiliations:** ^1^Department of Microbiology, Chonnam National University Medical School, Gwangju, South Korea; ^2^Department of Molecular Medicine (BK21plus), Chonnam National University Graduate School, Gwangju, South Korea; ^3^Department of Bioscience and Biotechnology, Hankuk University of Foreign Studies, Yongin, South Korea

**Keywords:** type 3 secretion system, *Salmonella* pathogenicity Island-1, synthetic biology, bacterial cancer therapy, targeted delivery, biotherapy

## Abstract

Targeted delivery of drugs is a key aspect of the successful treatment of serious conditions such as tumors. In the pursuit of accurate delivery with high specificity and low size limit for peptide drugs, a synthetic type 3 secretion system (T3SS) has been repurposed from a native genetic system encoded in *Salmonella* pathogenicity island-1 (SPI-1) with no virulence effectors. Here, we tested the potential of synthetic T3SS as drug delivery machinery for peptide-based drugs owing to its modular nature. First, the genetic system for synthetic T3SS was introduced into non-native host *E. coli*, which was chosen for its lack of *Salmonella*-driven virulence factors. Next, the mitochondrial targeting domain (MTD) of Noxa was tested as a cargo protein with anti-tumor activity. To this end, the gene encoding MTD was engineered for secretion through synthetic T3SS, thereby resulting in the tagged MTD at the N-terminus. When *E. coli* carrying synthetic T3SS and MTD on plasmids was administered into tumor-bearing mice, MTD with a secretion tag at the N-terminus was clearly detected in the tumor tissue after induction. Also, the tumor growth and mortality of tumor-bearing animals were mitigated by the cytotoxic activity of the delivered. Thus, this work potentiates the use of biotherapeutic bacteria for the treatment of tumors by implanting a dedicated delivery system.

## Introduction

Since Coley’s toxin was used in 1891, bacteria-based cancer therapy has been developed as a promising treatment (Kucerova and Cervinkova, 2016). Some bacteria have natural anti-tumor traits such as selectivity, cytotoxicity, and immunogenicity specifically against cancer and show persistence inside the body. Additionally, bacteria can carry therapeutic agents to tumors via engineered vectors (Song et al., 2018; Ashu et al., 2019; Duong et al., 2019). Only a few bacterial strains have been selected for cancer therapy with the aforementioned traits, including the genera *Salmonella*, *Lactobacillus*, *Listeria, Clostridium*, and *Escherichia coli* (Ashu et al., 2019; Duong et al., 2019; Sedighi et al., 2019). Studies have shown that facultative anaerobic bacteria such as *E. coli* and *Salmonella* selectively colonize and grow in tumors, especially in hypoxic and necrotic areas, thereby selectively delivering anti-tumor agents to these locations (Yu et al., 2004; Min et al., 2008; Jiang et al., 2010). Thus, numerous anti-tumor factors including therapeutic peptides have been packed into *E. coli* or *Salmonella* for delivery into hypoxic and necrotic areas of tumor site(s) (Jeong et al., 2014; Din et al., 2016; Lim et al., 2017; Zheng et al., 2017). Although the therapeutics are carried by tumor-targeting bacteria, a precise control of delivery has not been achieved, and is currently guided by the nature of the therapeutic itself, the signal peptides fused to protein drugs (Blight and Holland, 1994; Shokri et al., 2003), or nanoparticles (Luo et al., 2016). To improve the targeted delivery of peptide drugs, a phage-driven lysis system was employed wherein genes encoding bacterial cell lysis were encoded together with therapeutic molecules, thereby leading to the simultaneous release of the expressed therapeutics (Maratea et al., 1985; Miksch et al., 1997; Jain and Mekalanos, 2000). Therefore, the therapeutics were released from the tumor-targeting bacteria by activating a regulatable promoter, which resulted in an increased titer of peptide drugs and decreased tumor volume (Jeong et al., 2014; Lim et al., 2017). However, such a dramatic release could only sustain the anti-tumor effect for a short period of time due to the clearance of host bacteria after lysis.

Building a biological system with new entities has been refined with the help of synthetic biology. Consequently, a new system can be designed and constructed by genetic engineering using well-defined genetic parts for additional or repurposed functions. Moreover, the control of the system relies on the input(s) of the user rather than signals, as in a native system. Remarkable progress has been made in developing engineered systems such as complicated gene networks, thereby creating advanced materials, small molecule production, biopharmaceuticals production, and biotherapy (El Karoui et al., 2019; Wang and Zhang, 2019; Li et al., 2020; Zhou et al., 2020).

A *Salmonella* type 3 secretion system (T3SS) has been engineered for applications in biotechnology for the purification of peptides with high purity and titer (Widmaier et al., 2009; Singer et al., 2012; Azam et al., 2016; Metcalf and Tullman-Ercek, 2017). When compared to other secretion systems such as the Sec, Tat, and type 2 system, the advantages of the T3SS are direct translocation of peptides from bacterial cytosol to environment/host cytosol, secretion of multiple peptides, and maximum capacity for secretion substrate (∼233.5 kDa) (Radics et al., 2014; Wagner et al., 2018). However, the application of the native T3SS was limited by the tight regulation of expression at all levels (Bailly-Bechet et al., 2011; Deng et al., 2017; De Nisco et al., 2018). Recently, synthetic T3SS has been designed and built with a synthetic biology approach to overcome the hurdles where expression is unlinked to environmental cues or networks of the regulator. Unlike the native system, synthetic T3SS is controlled by a synthetic regulatory circuit, and therefore it is functional without limitations by environmental cues *in vitro*, while holding the unique features of T3SS (Song et al., 2017).

In this study, we tested the feasibility of the synthetic T3SS as a novel delivery system for peptide-based drugs. To this end, the mitochondrial targeting domain (MTD) of Noxa was chosen as a therapeutic cargo peptide. As a pro-death domain of Noxa involved in p53-induced cell death through mitochondrial dysfunction, the MTD peptide causes massive necrosis *in vitro* and in an animal model, thus resulting in tumor suppression (Seo et al., 2009; Morsi et al., 2018). Therefore, the MTD of Noxa was engineered for secretion through synthetic T3SS and tested for *in vitro* and *in vivo* anti-tumor activity. The cytotoxic activity of secreted MTD was confirmed when it was tested on cultured cancer cell lines. More importantly, MTD was detected in the tumor tissue and tumor regression was noted as a result of delivery of MTD through targeted *E. coli* equipped with synthetic T3SS. Thus, this work shows the potential of synthetic T3SS as a controllable delivery system, which in turn expands the boundaries of gene therapy via synthetic biology.

## Materials and Methods

### Bacterial Strains, Plasmids, and Culture Conditions

The *Escherichia coli* K-12 strain MG1655 was used as a host strain for the cultivation of the engineered *E. coli* strain. The MG1655 strain was transformed with plasmids encoding the controller and the refactored type 3 secretion system (Song et al., 2017), resulting in the creation of the EMU004 strain. The mitochondrial targeting domain (MTD) of human Noxa was amplified from the pLYS *P_BAD_::DS4.3-MTD* plasmid (Jeong et al., 2014) and introduced into ColE1 plasmid (EM_C095; Song et al., 2017) by Gibson assembly to generate pSL002. The MTD of Noxa was placed under the control of a constitutive promoter, P_J23110_, and the 167 N-terminal amino acids of SptP together with the cognate chaperone SicP at the 5’ end of SptP, located at the N-terminus of MTD on pSL002, served as a tag for secretion through the engineered T3SS. Then, pSL002 and EM_C095 was introduced into MG1655 and EMU004, respectively. The bacterial strains and plasmids are listed in Supplementary Table 1. A *Salmonella* strain with pLYS *P_BAD_::DS4.3-MTD* was used as a control for *in vitro* LDH assay.

Bacterial cultures were grown in L-broth (LB broth base; Duchefa # L1703.0500) at 37°C with vigorous aeration, unless indicated otherwise. All antibiotics were purchased from Sigma Chemicals and added at the following concentrations when necessary: ampicillin (#A0166; 50 μg ml^–1^), kanamycin (#K0879; 50 μg ml^–1^), spectinomycin (#S9137; 50 μg ml^–1^), and chloramphenicol (#C7795; 34 μg ml^–1^). For solid support medium, granulated agar (Duchefa #M1002.1000) was added as final 1.5 % (w/v). The engineered T3SS was induced by the addition of 10 μM isopropyl-β-D-1-thiogalactopyranoside (IPTG; Biosesang #367-93-1) and 10 μM *N*-(β-ketocaproyl-L-homoserine lactone (AHL; Cayman Chemical #10011207).

### Secretion Assay and Western Blot Analysis

Bacterial strains were cultured in L-broth at 37°C for 18 h and diluted 1:100 into fresh L-broth containing antibiotics. The culture was incubated at 37°C for 2 h at 200 rpm and then 10 μM of each inducer (IPTG, AHL) was added. The bacterial culture was further grown at 37°C for 6 h at 160 rpm. Culture supernatants were harvested by filtration through a 0.2-μm pore size filter unit after centrifugation at 15,000 *g* for 3 min and mixed with SDS loading buffer containing 0.2% β-mercaptoethanol. The pellet fraction was resuspended in PBS and prepared for western blot analysis as a control for the comparison of expression level. The supernatant or the pellet was run on 10% SDS-PAGE and the gels were transferred onto a PVDF membrane (GE Healthcare Life Science #A21504264). After being blocked in 5% skim milk (w/v of TBST) at RT for 1 h, the membrane was probed with 1:2,000 (v/v of 2.5% skim milk in TBST) diluted anti-Flag for SptP (Sigma Aldrich #MAB3118), anti-GroEL (Sigma Aldrich #G6532), or anti-Noxa for Noxa (abcam #13654) at RT for 1 h. The membranes were washed with TBST three times. For Flag-tagged SptP or Noxa, HRP-conjugated anti-mouse antibody (Thermo Fisher Scientific #62-6520) was used as a secondary antibody, but for the detection of GroEL as a cell lysis marker, HRP-conjugated anti-rabbit antibody (Thermo Fisher Scientific #A16110) was used as a secondary antibody. Each secondary antibody was diluted in 2.5% skim milk (1:4,000) and incubated at RT for 1 h. After washing the membrane, the signal was detected with a chemiluminescence substrate (Thermo Fisher Scientific #32209) on an iBright CL1500 Imaging system (Thermo Fisher Scientific).

### Cell Lines and Animal Experiments

A CT26 mouse colon carcinoma cell line and HeLa cells from the Waterborne Virus Bank (Seoul, South Korea) were used and grown in high-glucose Dulbecco’s Modified Eagle Medium containing 10% FBS and 1% penicillin-streptomycin (Well Gene #LS202-02). Cells were seeded at 5 × 10^5^ cells per ml for each assay. Female 6-week-old BALB/c mice were purchased from Samtako, South Korea. All animal care, experiments and euthanasia were performed in accordance with the protocols approved by the Chonnam National University Animal Research Committee (CNU IACUC-H-2019-14). Tumor-bearing mice were generated by subcutaneous implantation of CT26 tumor cells (10^6^ cells suspended in 30 μl PBS) into the right thigh. When the tumor sizes reached approximately 100–150 mm^3^, mice were injected with 1 × 10^8^ *E. coli* carrying engineered T3SS together with the MTD of Noxa (EMU007) through an intravenous route. The inducers for the engineered T3SS were administrated intraperitoneally 3 days post-infection (100 μM, respectively). Mice were sacrificed after anesthesia using a mixture of ketamine (200 mg kg^–1^) and xylazine (10 mg kg^–1^) for the determination of the delivered MTD.

### LDH Assay

The LDH (lactate dehydrogenase) released from cultured cell lines was determined using the LDH Cytotoxicity Assay Kit II (Abcam #ab65393) upon exposure to MTD. Briefly, the supernatant of bacterial culture was prepared as aforementioned from MG1655 carrying only MTD (EMU008), *E. coli* containing engineered T3SS and MTD (EMU007), or *Salmonella* strain carrying pLYS *P_BAD_::DS4.3-MTD* as a control with or without inducers. The supernatant was then concentrated using a centrifugal filter (Amicon Ultra 10K; Milipore #UFC901008) and the concentration of total fraction was measured by Bradford method. The indicated concentration of the supernatant from EMU007 with inducers was added to a HeLa cell culture in a 96-well plate. For the comparison of cytotoxicity of MTD in the engineered *Salmonella* or *E. coli*, 1 μg of each supernatant fraction was added to the HeLa or CT26 cell culture. After 16 h of culture at 37°C in a humidified chamber with 5% CO_2_, cultured cells were incubated with LDH lysis buffer for 30 s followed by centrifugation at 3,000 *g* for 5 min and the supernatant was analyzed following the manufacturer’s instructions. Absorbance readings of LDH reaction products were measured at 450 nm using a SpectraMax M5^®^ plate reader (Molecular Devices).

### Detection of Delivered MTD *in vivo* Tumor Model

A solid tumor was excised from each mouse 3 h after the addition of inducers and homogenized in 5 ml of PBS with 0.05% Triton X-100 (Polytron homogenizer; IKA, Ultra-Turrax T10). The homogenized tissues were centrifuged at 150 *g* for 10 min and the supernatant fraction was filtered through a 0.2-μm pore size filter unit to remove bacterial cells. The filtered fraction was analyzed by western blot analysis as mentioned above for the detection of MTD delivered by engineered *E. coli* and anti-β-actin antibody (sc-47778; Santa Cruz Technology) was used as a loading control for the assay.

### Statistical Analysis

Statistical analysis was performed using the Mann-Whitney *U* test for the LDH assay and two-tailed Student’s *t-*tests was used for the determination of tumor growth experiments. Survival was analyzed using the Kaplan-Meier curves and the log-rank test. *P* < 0.05 was considered significant for all analyses. All data are expressed as the mean ± SD.

## Results

### Functional Analysis of Engineered *Salmonella* T3SS in *E. coli*

**FIGURE 1:**
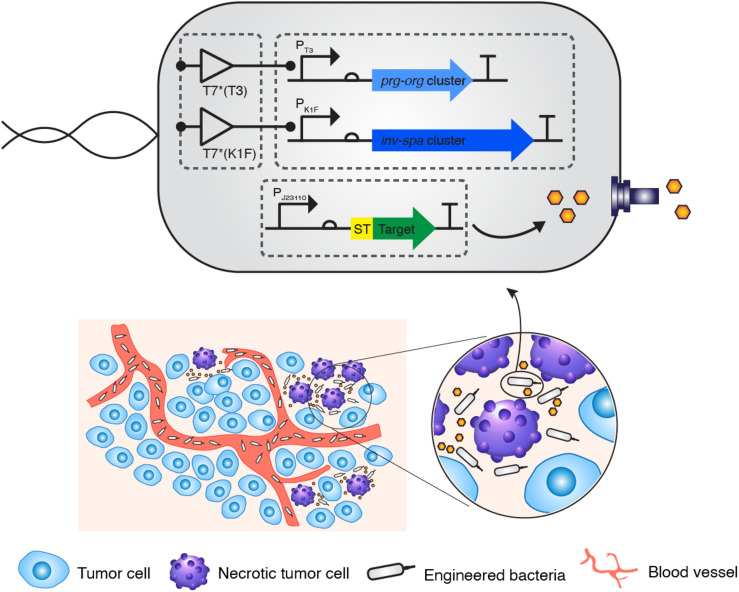
Strategic diagram of the delivery of therapeutic peptide into tumor tissue via bacteria with the synthetic type 3 secretion system. A minimal genetic system encoding the engineered type 3 secretion system was introduced into *E. coli* K-12 strain together with a controlling genetic circuit. When the “delivery agent” *E. coli* targets the necrotic area of a tumor, a therapeutic peptide is secreted into the region.

**FIGURE 2:**
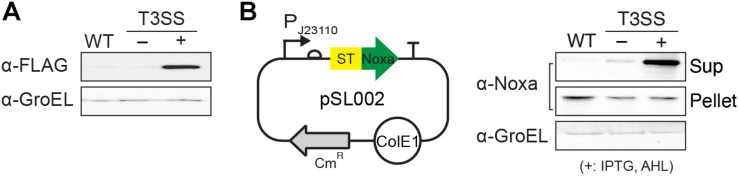
Function of enT3SS in non-native host, *E. coli*. **(A)** The secreted SptP was detected in the culture supernatant of *E. coli* containing plasmids for enT3SS and the reporter plasmid encoding the *Salmonella* effector protein, SptP. **(B)** A map of pSL002 encoding MTD with 167 amino acids of SptP at the N-terminus (left panel) and the representative western blot analysis of the secreted MTD in culture supernatant (right panel) (*n* = 3). The pellet fraction from each sample was also tested for the comparison of expression level (middle blot; pellet). WT indicates the results from MG1655 carrying only pSL002. enT3SS was induced by the addition of 10 μM of IPTG and AHL, respectively.

Synthetic T3SS was successfully engineered from SPI-1 for applications in biotechnology (Song et al., 2017). Unlike native T3SS, the function of synthetic T3SS is controlled by a genetic circuit rather than environmental cues in the native host. However, synthetic T3SS has not been applied and tested in any other bacteria for its modular function. Here, we implanted synthetic T3SS into a non-native host, *E. coli*, then tested it as a drug delivery system, wherein an antitumor peptide was expressed and secreted when administrated in a mouse tumor model (Figure 1). *E. coli* was chosen as a host strain for synthetic T3SS due to its low-toxicity, tumor-targeting ability, and high susceptibility to antibiotics in an animal (Min et al., 2008; Park et al., 2016; Supplementary Figure 1). First, all the plasmids encoding synthetic T3SS, hereinafter named enT3SS, were transformed into MG1655, which is the most widely used strain of *E. coli*. Next, plasmid containing the gene for the *Salmonella* effector, *sptP*, was also introduced into the *E. coli* equipped with enT3SS, the expression of which was under the control of a constitutive promoter (P_J23110_), and secretion was aided by a cognate chaperone on the same plasmid (EM_C095) (Song et al., 2017). When there was no enT3SS or two inputs for the control circuit, the secretion of SptP was not observed (Figure 2A, lanes 1 and 2). However, the secreted SptP was clearly detected in the supernatant upon the addition of inducers (Figure 2A, lane 3). Next, the *sptP* gene on the plasmid was replaced with the MTD of Noxa, where the MTD was placed after 167 aa of SptP, which served as a secretion tag (pSL002; Figure 2B; map). Indeed, the MTD was only detected in the culture supernatant of *E. coli* with induced enT3SS, regardless of the expression level (Figure 2B; western blot). The expressed amount of MTD in wild type *E. coli* was even 1.75-fold higher compared to that of *E. coli* with induced enT3SS in western blot analysis (pellet fraction in Figure 2B). Additionally, the growth retardation was not observed in *E. coli* with enT3SS upon induction (Supplementary Figure 2), and the secreted amount of MTD was approximately 7.322 (0.1 mgl^–1^ as measured by western blot analysis. These results suggest that enT3SS can be applied for the secretion of proteins with the help of the designated tag at the N-terminus.

### Measurement of the Cytotoxic Activity of Secreted MTD of Noxa Using LDH Assay

**FIGURE 3:**
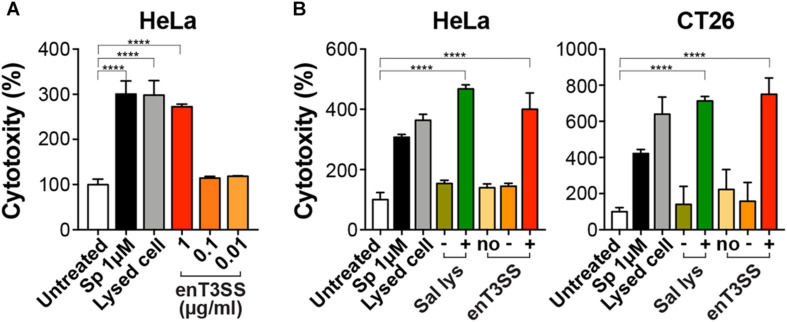
Cytotoxic activity of MTD with secretion tag. The cytotoxicity of the secreted MTD was measured by LDH assay using cancer cell lines. The percent cytotoxicity was calculated using the untreated cells as negative control. As positive control, cells were treated with staurosporine (Sp; 1 μM) or lysis buffer. **(A)** HeLa cells were treated with the indicated concentration of the supernatant fraction from *E. coli* with enT3SS (EMU007) after induction with 10 μM of IPTG and AHL, respectively. Significance is indicated as ****P < 0.0001 (*n* = 8). **(B)** One μg of the supernatant fraction from EMU008 (no enT3SS; no) or EMU007 was added to the culture of HeLa or CT26 cells without (−) or with induction (+). For comparison, the culture supernatant of *Salmonella* carrying pLYS *P_BAD_::DS4.3-MTD* (Sal lys) was also tested in both cell cultures with (+; 0.04 % arabinose) or without induction (−). Significance is shown as ****P < 0.0001 (*n* = 9 for HeLa; *n* = 7 for CT26 cells).

Next, we evaluated the activity of MTD with an SptP-secretion tag at the N-terminus using cultured tumor cell lines. Cytotoxic activity was measured by LDH release assay upon the treatment of the tumor cells with the supernatant fraction from the *E. coli* culture carrying pSL002 with induced enT3SS. When the supernatant fraction was applied to HeLa cells, the cytotoxic effect increased in proportion to the concentration of supernatant. The maximum cytotoxicity of MTD was achieved with 1 μg of the supernatant fraction, that is, an increase in cytotoxicity of approximately 280% in comparison with untreated cells (Figure 3A). Also, increased cytotoxicity was also observed in CT26 cells, similar to HeLa cells, upon treatment of 1 μg of supernatant fraction from the bacterial culture with induced enT3SS (Figure 3B). Moreover, the cytotoxicity was as high as the MTD conjugated with cell-penetrating peptide released from *Salmonella* by a phage-driven lysis system (Jeong et al., 2014). After treatment with the supernatant fraction of *E. coli* culture containing MTD and induced enT3SS, the MTD with SptP tag was detected from the whole cell lysates of HeLa cells by western blot analysis (Supplementary Figure 3). Altogether, the secreted MTD retained activity against tumor cells in the presence of the secretion tag.

### Determination of Anti-tumor Effect of Secreted MTD in Murine Tumor Model

**FIGURE 4:**
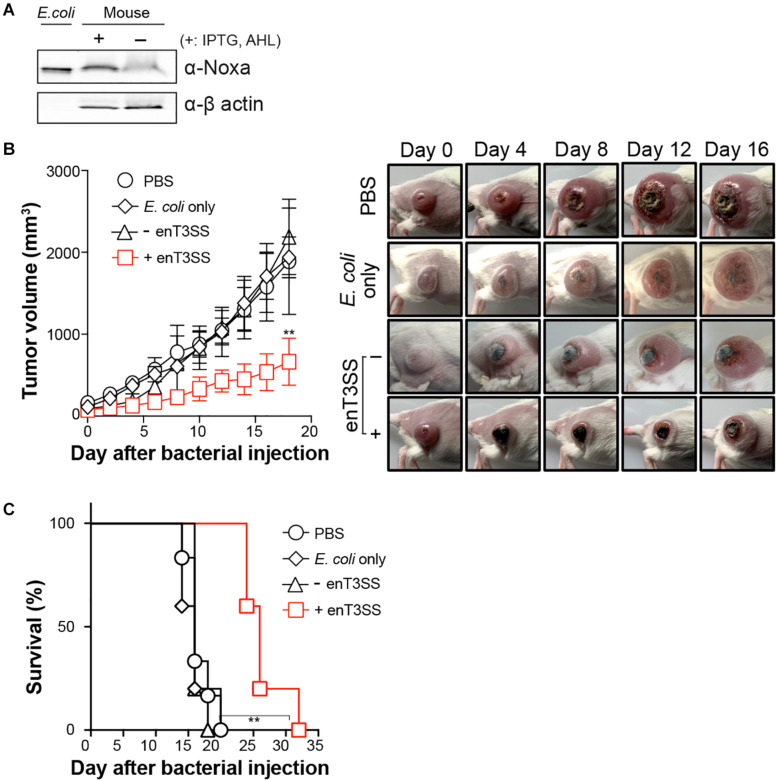
Tumor regression by MTD delivered through enT3SS loaded on *E. coli*. CT26 tumor-bearing mice were intravenously injected with *E. coli* carrying MTD and enT3SS (EMU007) and inducers were administrated 3 days post infection. *E. coli* carrying only MTD (EMU008) was also administrated as a control. **(A)** Western blot analysis of tumor tissue using anti-Noxa antibody (representative blot). The supernatant of bacterial culture (lane 1; *E. coli*) or β-actin (bottom blot) was used as a control for the assay. **(B)** The measurement of tumor size (left) and representative pictures of mice (right). Significance is indicated as **P = 0.0016 (*n* = 5 per group). **(C)** The percent survival of tumor-bearing mice administrated with *E. coli* carrying enT3SS (EMU007; +) or no enT3SS (EMU008). Significance is indicated as **P = 0.0012 (*n* = 5 per group).

Since the cytotoxicity of the secreted MTD was confirmed *in vitro*, we then tested its anti-tumor activity in a murine tumor model. CT26 tumor-bearing BALB/c mice were administered engineered *E. coli* through an intravenous route. Since *E. coli* is usually targeted to tumor tissue 3 days after inoculation (Min et al., 2008), the inducers were administered 3 days post-infection. First, the secreted MTD was confirmed from the excised tumor tissue 3 h after the injection of the inducers (Figure 4A). Additionally, the tumor suppression effect was further checked by the measurement of tumor growth after the introduction of the engineered bacteria (Figure 4B). Without treatment, the tumor size gradually increased and eventually all mice died or were sacrificed when the size reached 1,500 mm^3^. Previous studies showed that the tumor growth was approximately 1.2-fold suppressed by targeted *E. coli* itself or by *E. coli* carrying a protein with no anti-tumor activity (Min et al., 2008; Forbes, 2010; Van Dessel et al., 2015). However, the tumor growth rate decreased when the MTD was secreted from the bacteria within the tumor tissue and the average tumor size was 2.85 times smaller than that in a PBS-treated sample on the last day. Also, the percent survival clearly increased from 20 days without treatment to 32 days in mice with the MTD delivered into tumor tissue via *E. coli* with induced enT3SS.

## Discussion

Precisely controlled delivery is one of the keys to successful treatment with biologic drugs. Certain bacterial species including *E. coli* can target and colonize solid tumors, often leading to the retardation of tumor growth or even tumor clearance (Song et al., 2018). Therefore, anti-tumor factors have been loaded on such bacteria for delivery, and for enhancement of the oncolytic effect of the bacteria itself (Jeong et al., 2014; Din et al., 2016; Lim et al., 2017; Zheng et al., 2017). However, even highly expressed factors often fail to be delivered due to the lack of a delivery system, and rely on release from lysed host bacteria or the periplasmic space of bacteria, which has low efficiency (Jeong et al., 2014; Lim et al., 2017). In this study, we tested the feasibility of an engineered bacterial secretion system, synthetic T3SS, as a delivery machine for targeted tumor therapy. When synthetic T3SS was introduced in *E. coli*, the target proteins were efficiently secreted through this system (Figure 2). Since the expression of target protein was placed under the control of a constitutive promoter, the activity was dependent on the amount of secretion, which is regulated by a two input-genetic circuits for the control of synthetic T3SS (Figure 1). Furthermore, the cytotoxicity of MTD against tumor cell line HeLa and CT26 was sustained even in the presence of a secretion tag at the N-terminus. A secretion tag composed of 167 amino acids from N-terminus of *Salmonella* SptP can presumably either aid the penetration of MTD or change the properties of the eukaryotic host membrane. The activity of MTD with a secretion tag was shown to be as effective as MTD fused to DS4.3, a cell-penetrating peptide (Jeong et al., 2014; Figure 3). As a matter of fact, the MTD was identified in target cells after the treatment with supernatant fraction containing MTD with secretion tag (Supplementary Figure 2). Nevertheless, the role of an SptP tag should be elucidated in future studies for better understanding of the delivery system. This may be done by using reporters such as luciferase, iLOV variants, or a split fluorescent protein wherein the tagged reporter protein can be secreted through T3SS and live imaging can be performed. Finally, the anti-tumor activity of tagged MTD was verified in tumor-bearing mice. When MTD was delivered to tumor tissues by enT3SS, the tumor volume decreased and the survival of tumor-bearing mice increased in comparison with the mice treated with PBS or *E. coli* only (Figure 4). However, the tumor was not completely cleared and mice also could not survive from CT26-driven cancer. This incomplete clearance can be explained by the loss of plasmids over time (Supplementary Figure 4). In a mouse model, only 20 % of the bacteria hold plasmids within tumor tissues at day 10. This obstacle can be overcome by the stabilizing the genetic system of enT3SS through chromosomal integration of encoding gene clusters. Collectively, the synthetic T3SS enables the delivery of therapeutic peptides via a controllable synthetic system, that can easily be revolutionized by genetic manipulation.

## Data Availability Statement

The raw data supporting the conclusions of this article will be made available by the authors, without undue reservation.

## Ethics Statement

The animal study was reviewed and approved by the Chonnam National University Animal Research Committee (CNU IACUC-H-2019-14).

## Author Contributions

DL, J-HJ, and MS designed the experiments. DL, WJ, and MS performed the experiments. DL and MS discussed the data and wrote the manuscript. All authors contributed to the article and approved the submitted version.

## Conflict of Interest

The authors declare that the research was conducted in the absence of any commercial or financial relationships that could be construed as a potential conflict of interest.
