# Supplementary material for: Targeted Delivery of the Mitochondrial Target Domain of Noxa to Tumor Tissue via Synthetic Secretion System in E. coli
Source: Front Bioeng Biotechnol. 2020 Jul 17;8:840. doi: 10.3389/fbioe.2020.00840 (PMC7379172; doi:10.3389/fbioe.2020.00840)
Supplement: Supplementary file 1 [file Table_1.docx]

Supplementary Table 1. Bacterial strains and plasmids

| Strains | Genotype | References |
| --- | --- | --- |
| MG1655 | *Escherichia coli* K12 |  |
| EMU004 | MG1655, enT3SS^*^ (pMR097, pMR108, pMR061) | This study |
| EMU007 | MG1655, enT3SS (pMR097, pMR108, pMR061), pSL002 | This study |
| EMU006 | MG1655, enT3SS (pMR097, pMR108, pMR061), EM_C095 | This study |
| EMU008 | MG1655, pSL002 | This study |
| 14028s | *Salmonella typhimurium* |  |
| Plasmids |  |  |
| pMR097 | Same as pMR_prgorgR5 | Song et al., 2017 |
| pMR108 | Same as pMR_invspaR4 | Song et al., 2017 |
| pMR061 | Same as pMR_controller3 | Song et al., 2017 |
| EM_C095 | Same as pMR_reporter | Song et al., 2017 |
| pLYS *P_BAD_::DS4.3-MTD* |  | Jeong et al., 2014 |
| pSL002 | P_J23110_::sicP::sptP tag::Noxa MTD | This study |

* enT3SS indicates the plasmids encoding structural component of the engineered T3SS (pMR097 and pMR108) and control circuit (pMR061).

**
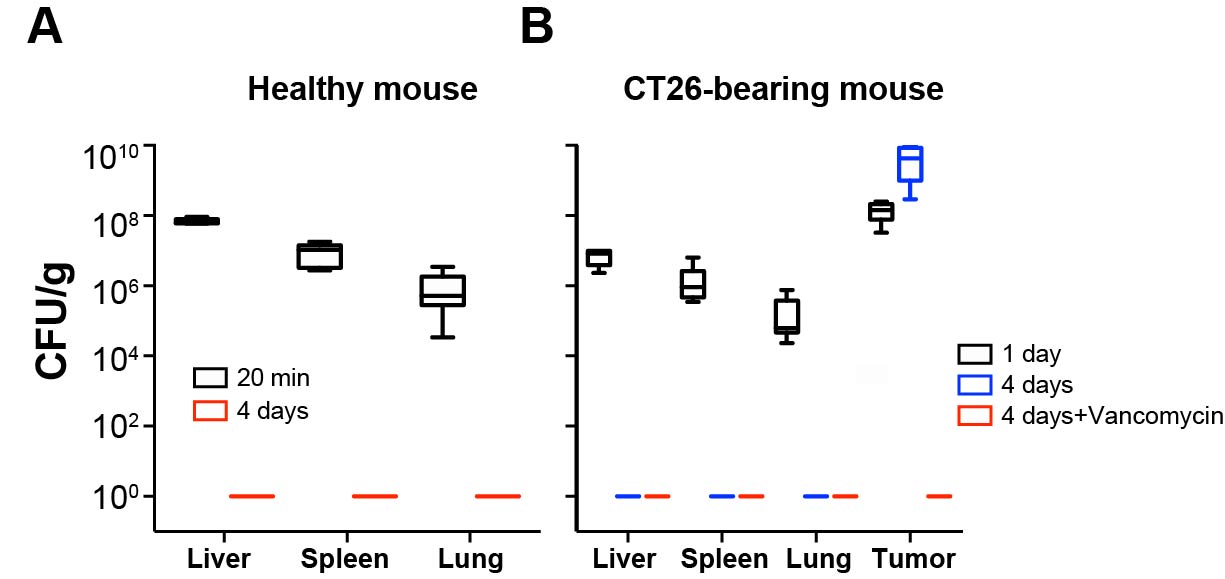
**

**Supplementary Figure 1. Determination of bacterial loads and the clearance of tumor-targeted *E. coli* in animal model.**

(A) Organ distribution of healthy mice intravenously injected with *E. coli* (1x10^8^ CFU/mouse). Each organ was isolated aseptically at the indicated time (20 min and 4 days post-infection), homogenized, and plated on LB agar plate to enumerate the bacterial load (n=6 for 20 min; n=7 for 4 days). (B) WT *E. coli* (MG1655) was administered into CT26 tumor-bearing mice through intravenous injection (1x10^8^ CFU/mouse). At 1 (black) and 4 (blue) days post-infection, each organ was isolated and analyzed for organ distribution of injected bacteria (n=6 for 1 day; n=7 for 4 days post-infection). For the elimination of the targeted *E. coli* from mice, vancomycin (30 mg kg^-1^; Sigma Chemicals) was administered intraperitoneally every 6 hr three times at 3 days post-infection (red) and organs were isolated at 4 days post-infection. To enumerate the residual bacteria, the homogenized organ was plated on LB agar individually (n=7).


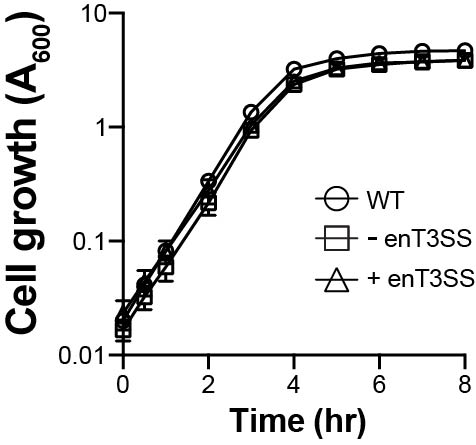


**Supplementary Figure 2. Growth curve of *E. coli* with enT3SS.** *E. coli* carrying pSL002 encoding MTD only was used as a control (WT, open circle) and *E. coli* carrying pSL002 together with enT3SS was uninduced (-, open square) or induced (+, open triangle) by the addition of 10 μM IPTG and AHL, respectively.


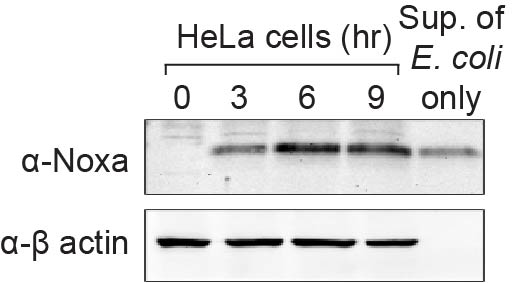


**Supplementary Figure 3. Detection of the delivered MTD in HeLa cells.** HeLa cell culture was treated with 1 μg of culture supernatant fraction of *E. coli* culture carrying pSL002 together with the induced enT3SS. At indicated time points (0, 3, 6, 9 hr), the treated HeLa cell culture was washed twice with 1x PBS and total cell lysate was harvested by centrifugation after lysis with the RIPA buffer (Thermo Fisher Scientific #89900). The MTD of Noxa in HeLa cell lysates were detected by western blot analysis using anti-Noxa antibody. The supernatant fraction used for the treatment was used as a control for Noxa (lane5). Beta-actin was only detected from HeLa cell lysates, which served as a loading control for each lysate.


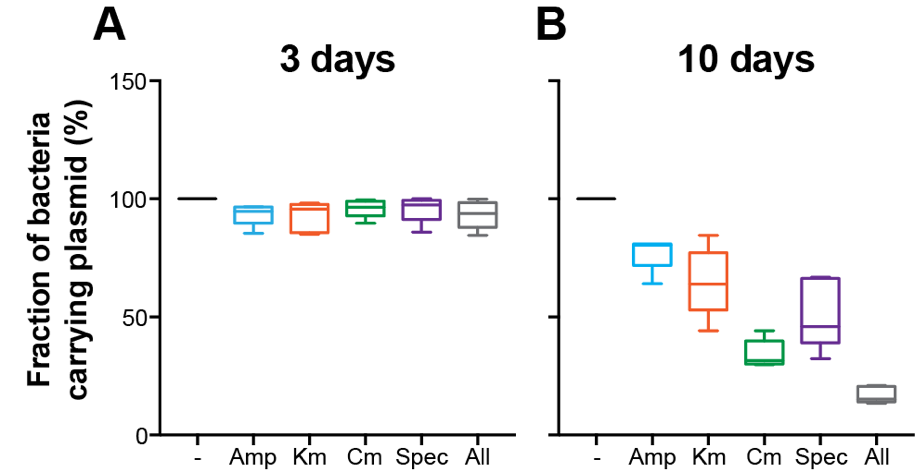


**Supplementary Figure 4. Enumeration of bacterial numbers carrying plasmid(s) inside CT26 tumor tissue.** *E. coli* carrying plasmids encoding enT3SS and MTD was intravenously injected into CT26 tumor-bearing mice. Tumor tissues were excised at 3 (A) and 10 days after injection and bacteria was isolated from the homogenized tumor tissues. The isolated bacteria were then plated on LB plates containing no drug (black), ampicillin (blue; 50 μg ml^-1^), kanamycin (orange; 50 μg ml^-1^), chloramphenicol (green; 34 μg ml^-1^) or spectinomycin (purple; 50 μg ml^-1^), and all four antibiotics for the estimation of CFU (n=.
